# Kalman estimator- and general linear model-based on-line brain activation mapping by near-infrared spectroscopy

**DOI:** 10.1186/1475-925X-9-82

**Published:** 2010-12-08

**Authors:** Xiao-Su Hu, Keum-Shik Hong, Shuzhi S Ge, Myung-Yung Jeong

**Affiliations:** 1 Department of Cogno-Mechatronics Engineering, Pusan National University; 30 Jangjeon-dong, Geumjeong-gu, Busan609-735, Korea; 2 Department of Cogno-Mechatronics Engineering and School of Mechanical Engineering, Pusan National University; 30 Jangjeon-dong, Geumjeong-gu, Busan 609-735, Korea; 3 Department of Electrical and Computer Engineering, The National University of Singapore, Singapore 117576

## Abstract

**Background:**

Near-infrared spectroscopy (NIRS) is a non-invasive neuroimaging technique that recently has been developed to measure the changes of cerebral blood oxygenation associated with brain activities. To date, for functional brain mapping applications, there is no standard on-line method for analysing NIRS data.

**Methods:**

In this paper, a novel on-line NIRS data analysis framework taking advantages of both the general linear model (GLM) and the Kalman estimator is devised. The Kalman estimator is used to update the GLM coefficients recursively, and one critical coefficient regarding brain activities is then passed to a *t*-statistical test. The *t*-statistical test result is used to update a topographic brain activation map. Meanwhile, a set of high-pass filters is plugged into the GLM to prevent very low-frequency noises, and an autoregressive (AR) model is used to prevent the temporal correlation caused by physiological noises in NIRS time series. A set of data recorded in finger tapping experiments is studied using the proposed framework.

**Results:**

The obtained results suggest that the method can effectively track the task related brain activation areas, and prevent the noise distortion in the estimation while the experiment is running. Thereby, the potential of the proposed method for real-time NIRS-based brain imaging was demonstrated.

**Conclusions:**

This paper presents a novel on-line approach for analysing NIRS data for functional brain mapping applications. This approach demonstrates the potential of a real-time-updating topographic brain activation map.

## Background

Near-infrared spectroscopy (NIRS), an emerging brain imaging technique, measures the hemodynamic changes that effectively reflect the brain activity occurring while people perform a wide range of mental tasks [1-5]. It can provide both topographic [2,4,6] and tomographic [1,7] brain images. Specifically, NIRS monitors the regional cerebral blood flow (rCBF) variation by measuring, through the skull, the absorption changes of near-infrared light at wavelengths between 650 nm and 950 nm [3]. These changes are caused by the concentration variations of oxy-hemoglobin (HbO) and deoxy-hemoglobin (HbR), two primary absorbing chromophores in brain capillary blood.

NIRS, compared with other prevalent brain imaging and activity measurement techniques such as electroencephalography (EEG) and functional magnetic resonance imaging (fMRI), offers itself as a trade-off between spatial and temporal resolutions. The usability and drawbacks of NIRS methods, as discussed in a detailed review and comparison with other neuroimaging methods, was provided by Perrey [6]. fMRI has been used over the past decade in a growing number of applications. The critical drawbacks of the fMRI-based approaches, however, are the cost and the non-portability of the fMRI scanner. In fact, another comprehensive review [8], in comparing the respective features of NIRS and fMRI, concluded that NIRS has great potentials for neurological and psychiatric applications, due to its simplicity, portability, and insensitivity to motion artifacts. Meanwhile, the EEG technique is limited, due to its poor spatial resolution and low signal-to-noise ratios in many applications; NIRS can provide comparatively better quality in these aspects [9]. Indeed NIRS, in its wide applicability, might help to bring functional imaging to the patient's bedside [3].

## Methods

There is currently no standard method of topographic NIRS data analysis for brain mapping. In NIRS detection of hemodynamic responses, the light attenuation measured by the equipment needs to be converted to HbO and HbR concentration changes via the modified Beer-Lambert law (MBLL) [10]. Hence, a differential path length factor (DPF, intra- and inter-subject varying) in the MBLL has to be assumed to account for the increase of the path length between a source and a detector [11].

The classical approach in topographic NIRS data analysis is a paired *t*-test to determine if a concentration change between two states (for instance, "rest" vs. "task") is statistically significant. Many researchers nowadays use this approach [12-14], because it is simple and, thus, can provide a quick assessment to the task. One of the most popular tools in this regard is a Matlab-based program known as HomER ([15]http://www.nmr.mgh.harvard.edu/PMI/).

However, there are limitations to the classical *t*-test. First, a maximum activation period needs to be predefined. The remaining temporal information not included in the defined activation period therefore is ignored, leading to underestimation of brain activation. Another problem is the DPF assumption. Since the DPF is intra- and inter-subject variant and impossible to be measured for every measurement location with commonly obtainable continuous wave NIRS equipment, use of a constant DPF leads to biased estimation of concentration changes [16].

To overcome such problems of the classical *t*-test, a number of research groups have used various general linear model (GLM)-based methods for analysis of NIRS data [16-20]. The GLM-based methods were initially developed for fMRI-based functional brain mapping [21]. The GLM is a statistical linear concept that explains measured data in the form of a linear combination of several explanatory variables plus an error term. The explanatory variables, modelled according to the time course, separately account for the brain-activity-evoked signals and noises. Therefore, the estimation of brain activity is reduced to the problem of estimating the relevant coefficients with proper statistics.

The GLM-based methods negate the need for user-defined rest and task periods, because the response is modelled according to the entire time course. The temporal information over the entire time course, thus, is examined. On the other hand, the GLM-based methods investigate the temporal variation pattern of the signal, and estimate the coefficients with statistics at different measurement locations, separately. Therefore, these methods are robust in cases where an assumed constant DPF is used.

GLM-based methods, however, cannot offer on-line analysis. This constrains their use in applications where real-time information or feedback is required. Real-time brain imaging data analysis in comparison with off-line methods, significantly, improves the information acquisition rate and the feedback speed. Furthermore, it may potentially benefit the development of brain-computer interfaces (BCI).

The critical task in modifying a GLM-based method on-line is the recursive estimation of GLM coefficients. Previous researchers have used different methods to achieve recursive estimation, including recursive least square [22], Cholesky-decomposition-based recursive least square [23], and Kalman filtering [24,25]. All these approaches can effectively and recursively estimate GLM coefficients.

It is not sufficient to draw an updated brain activation map only by estimating GLM coefficients on-line. It is known that NIRS time series contain noises from different sources. Very-low-frequency noises caused by optodes shifts or slow cardiac/vascular artifacts [26], for example, might lead to biased estimation. The temporal correlation caused by physiological (cardiac, respiratory, blood pressure) noises might lead to an inflated *t*-value and, thereby, overestimation of brain activation. Furthermore, for functional brain mapping applications, a statistical test is very important, since it will provide significance verification of the derived estimation. All these issues with regard to on-line versions of GLM-based methods need to be addressed.

On-line estimation of GLM coefficients is generally discussed in [24]. In [25], the whole framework provided on-line estimation of GLM coefficients, and a relevant statistical test analysed the fMRI data. However, this work did not consider temporal correlation in fMRI data, which might also exist in NIRS data. In [26], a framework for NIRS-based BCI applications was developed that can estimate GLM coefficients without statistical information in classifying different hand tasks in real-time. In [27], the feasibility of estimating GLM coefficients on-line using a Kalman filter by studying a set of fMRI data was examined.

In the present study, we develop an on-line Kalman-estimator- and GLM-based NIRS data processing framework for brain activation mapping. We aim to answer two questions. (i) Is it possible to covert the off-line GLM-based method to an on-line version for NIRS-based brain activation mapping? The proposed method can provide updated brain activation maps on-line as well as track task-related brain-active areas from an early stage while the experiment is running. (ii) Can the proposed method prevent noises that distort the estimation while data is sequentially incorporated?

### NIRS measurement system and experimental procedure

**Figure 1:**
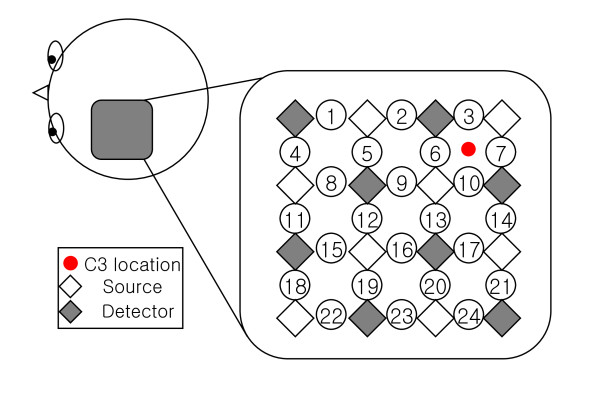
**Channel distribution and measurement location on head**. The detected area covers the primary motor cortex, dorsolateral prefrontal cortex, and Broca's area of the left hemisphere of the subjects. The C3 location, in the international 10-20 system, is used as a reference.

Five right-handed healthy volunteers (all male, aged 24 to 34 years) participated in the experiment. None of the participants had a history of any neurological disorder. All of the participants provided written informed consent. The experiment was conducted in accordance with the latest Declaration of Helsinki. The data were acquired with a continuous-wave NIRS imaging system (DYNOT: DYnamic Near-infrared Optical Tomography) obtained from NIRx Medical Technologies, Brooklyn, NY, at a sampling rate of 1.81 Hz. The system emits laser lights of different wavelengths (760 nm and 830 nm) from each source. Figure 1 shows the channel distribution and measurement location. The distance between different optodes is 2 mm.

In the experiment, the subjects were asked to perform a finger-tapping task. The experiment consisted of a 42 sec preparation period and 10 sessions. Each session included a 21 sec finger-tapping period and a 30 sec rest period. Accordingly, the total duration of the experiment was 552 sec.

### Analysis framework of NIRS data

**Figure 2:**
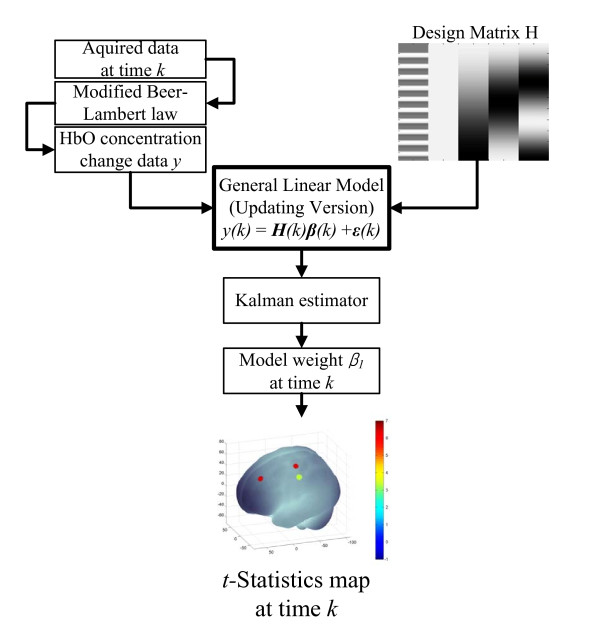
**Schematic flow chart of the framework**.

A schematic summarization of our method is as follows. (i) The relative concentration changes of two blood chromospheres, HbO and HbR, are calculated via MBLL [10,27]; (ii) A linear model is built according to the experimental procedure to fit the relative HbO concentration change. The model describes both the signals corresponding to the brain activity and the noise; (iii) The model coefficients are recursively estimated using the Kalman estimator; (iv) At every time step, the brain-activity-related coefficient is selected, and then passed to the *t*-statistical test to determine if its value is statistically greater than zero (a value greater than zero indicates brain activity): In this way, a probability brain activation map is drawn. Figure 2 is a schematic flow chart of the framework.

### NIRS measurement model

In NIRS measurement, the optical density variation (Δ*OD*) can be expressed as a linear combination of hemoglobin concentration changes (Δ*C_HbO _*and Δ*C_HbR_*) multiplied by proper coefficients. Their relationship is described in MBLL terms as

(1) $$\Delta OD^{\lambda ,i}=\alpha^{\lambda }\Delta C\;L^{\lambda ,i}DPF^{\lambda }$$

where $\alpha^{\lambda }\Delta C=\alpha_{HbO}^{\lambda }\Delta C_{HbO}+\alpha_{HbR}^{\lambda }\Delta C_{HbR}$, *λ *is the wavelength of the laser source, *i *indicates channel number, *α_HbO _*[μM^-1^mm^-1^] and *α_HbR _*[μM^-1^mm^-1^] are the extinction coefficients of the HbO and HbR, *L *is the distance between the source and the detector, and DPF is the differential path length factor. In the present study, the optical density variation was derived by dividing the light intensity measured at each time step by the light intensity measured at the first time step.

### General linear model (GLM)

In NIRS-based studies, both HbO and HbR concentration changes can reflect changes in the rCBF. However, it has been suggested that HbO is a more sensitive indicator of such changes [28]. Therefore, only the HbO concentration change data was considered in the present study.

The GLM design process is described in detail in [29]; we provide only a brief description here. A design matrix *H *including a set of explanatory variables is predefined in order to model the observed NIRS time series. Five explanatory variables are considered. The first variable models the HbO concentration changes (the brain activity signals) using a stimulus vector convolved with the basis function (BF, a double-gamma model; [30]). The second one models the baseline level, and the remaining variables represent a set of high-pass filters (discrete cosine transform, DCT) [30] with a cut-off frequency of 0.0006 Hz.

At time *k*, *y^i^*(*k*), the measured NIRS data of channel *i*, is predicted by *H*(*k*), specifically by multiplying a coefficient vector plus an error term *ε^i^*(*k*). The model can then be expressed as

(2) $$y^{i}(k)=H(k)\beta^{i}(k)+\varepsilon^{i}(k)$$

where *β^i^*(*k*) is the coefficient vector quantifying the magnitude of the explanatory variables. In vector *β^i^*(*k*), we are interested in the component *β^i^*_1_(*k*), which reflects the magnitude of the task-evoked brain response: By statistically determining if it is greater than zero, the existence of brain activity at the area covered by channel *i *can be confirmed (it is greater than zero) or ruled out (it is less than zero).

Several physiological processes are known to produce temporal correlation in NIRS data, which might lead to inflated *t*-values, and thus underestimation of brain activity. One way to deal with this problem is to make the model fit an AR (*p*) model (an autoregressive model of the order *p*). This leads to a decomposition of the error term *ε *into a systematic and a model conform error part. After this, the AR transformation coefficient is applied to both sides of the regression equation

(3) $$y^{i}(k)\text{-}\rho y^{i}(k\text{-}1)=H(k)\text{-}\rho H(k\text{-}1)\beta^{i}(k)+u^{i}(k)$$

where *ρ *is the estimated autocorrelation coefficient in an AR(1) process, and *u*(*k*) = *ρε*(*k-1*) + *ε*(*k*). By redefining each transformed variable, that is, y^i^*(*k*) = *y^i^*(*k*) - *ρy^i^*(*k-*1), H*(*k*) = *H*(*k*) - *ρH*(*k-*1), one can simplify Equation (3) to

(4) $$y^{i*}(k)=H^{*}(k)\beta^{i}(k)+u^{i}(k)$$

It is worth noting that we make an assumption, |*β*(*k*) - *β*(*k-*1)| <*ζ*, for the AR(1) model used on-line in the current study, where *ζ *> 0 is an arbitrary small number. This assumption compromises the model's robustness. However, as the result shows, the temporal correlation can be effectively reduced. In the present study, *β^i^*(*k*) was updated with a Kalman estimator.

### Kalman estimator

The Kalman filtering method is a recursive tracking scheme that estimates the state of a process using an updated regularized linear inversion routine [31]. After decades of development, the Kalman filtering is very mature. Due to its remarkable estimation performance, the Kalman filtering is widely used in many areas [32-34] including neuroscience [24,25,35,36]. In the present study, the Kalman filter was used as a model coefficients estimator. The model coefficients from all of the 24 channels were updated in parallel. For a given channel, the state vector, transition equation and observation equation can be described in the form

(5) $$X(k)={\left[\begin{array}{cccc} \beta_{1}(k) & \beta_{2}(k) & ... & \beta_{L}(k) \end{array}\right]}^{T}$$

(6) $$X(k)=A\hat{X}(k-1)+w(k)$$

(7) y(k)=H(k)X(k)+v(k)

where *L *is the number of explanatory variables. The state is assumed to follow a random walk with zero drift over time: Thus, *A *equals the identity matrix, and the process noise *w*(*k*) ~ *N*(0, *Q*), *y*(*k*) is the measured data, *H*(*k*) is the vector of explanatory variables, and the observation noise *v*(*k*) ~ *N*(0, *R*). The filter performs state estimation by the iterative process

(8) $${\hat{X}}^{-}(k)=A\hat{X}(k-1)$$

(9) $$P^{-}(k)=AP(k-1)A^{T}+Q$$

(10) $$K(k)=P^{-}(k)H^{T}(k)E^{-1}(k)$$

(11) $$\hat{X}(k)={\hat{X}}^{-}(k)+K(k)\Delta y(k)$$

(12) $$P(k)=(I-K(k)H(k))P^{-}(k),$$

where *E*(*k*) = *H*(*k*)*P*^-^(*k*)*H^T^*+*R*, $\Delta y(k)=y(k)-H(k){\hat{X}}_{}^{-}(k)$, *K*(*k*) is the Kalman gain, and *P*(*k*) is the updated error covariance matrix. In this notation, the superscript (-) refers to the intermediate state and covariance predictions provided by the state update model, which are then modified by the measured data to produce the next state value. In the present study, the state vector was initialized to zero. The a priori estimates of the process and observation noise covariances (*Q *and *R *respectively) were (1%/sec)^2 ^and (0.5 μM/sec)^2^, according to a restricted maximum likelihood (ReML) estimation and an empirical-experimental performance check based on a set of training data. We collected the training data during 3 sessions of finger tapping for each subject. We estimated the *Q *and *R *values in two steps: They were estimated separately from each of the subjects by ReML, averaged, and then adjusted according to the performance in practically estimation based on the training data.

### *t*-Statistics

The estimated model coefficient vector *β *was used to calculate a relevant *t*-value for a one-tailed *t*-test to test the null hypothesis *c^T ^β *= 0 [29]. In the present study, the *t*-statistics of channel *i *at time step *k *were obtained using

(13) $$t^{i}(k)=\frac{c^{T}\beta^{i}(k)}{\sqrt{{\hat{\sigma }}^{i2}(k)c^{T}{\left[\sum_{\text{1}}^{k}H_{k}^{T}H_{k}\right]}^{-\text{1}}c}},$$

where *c *is a vector of contrast for selecting the coefficient of interest [29], and ${\hat{\sigma }}^{i2}$ is the residual sum-of-squares divided by the appropriate degrees of freedom, and is given by

(14) $${\hat{\sigma }}^{i2}(k)=\frac{1}{k-L}\sum_{1}^{k}{\left[y_{k}^{i}-H_{k}\beta_{k}^{i}\right]}^{2},$$

where *L *is the number of regressors. Therefore, the null hypothesis *c^T ^β_i_*(*k*) = 0 was assessed by comparing *t^i^*(*k*) with a *t*-distribution with *k-L *degrees of freedom. By setting proper *p*-values with Bonferroni correction, a statistical activation map of the detected area could be displayed.

## Results

To simulate a real-time process, each measured data was incorporated sequentially in the analysis and updated at each time step. The entire procedure was simulated with Matlab at this stage. The computation time for one processing step was approximately 0.015 ± 0.0025 sec (mean ± standard deviation, 4 subjects averaged).

**Figure 3:**
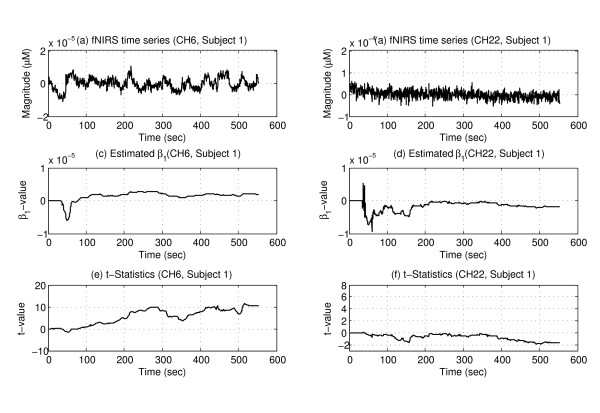
**Representative plots of different quantities estimated on-line**. Panels (a) and (b) show the NIRS time series measured from the representative channels. Panels (c) and (d) show separately the estimated critical model coefficient *β_1_*. Panels (e) and (f) show separately the calculated *t*-statistics.

Figure 3 depicts the raw data, and the estimated and t-statistics values of two representative channels from subject 1: the activated area (channel 6) and an inactivated area (channel 22). The raw NIRS time series are plotted in the top panels. The time evolutions of the estimated *β*_1 _are plotted in the middle panels. The corresponding *t*-statistics are plotted in the bottom panels. These values were estimated after the 6th sampling.

**Figure 4:**
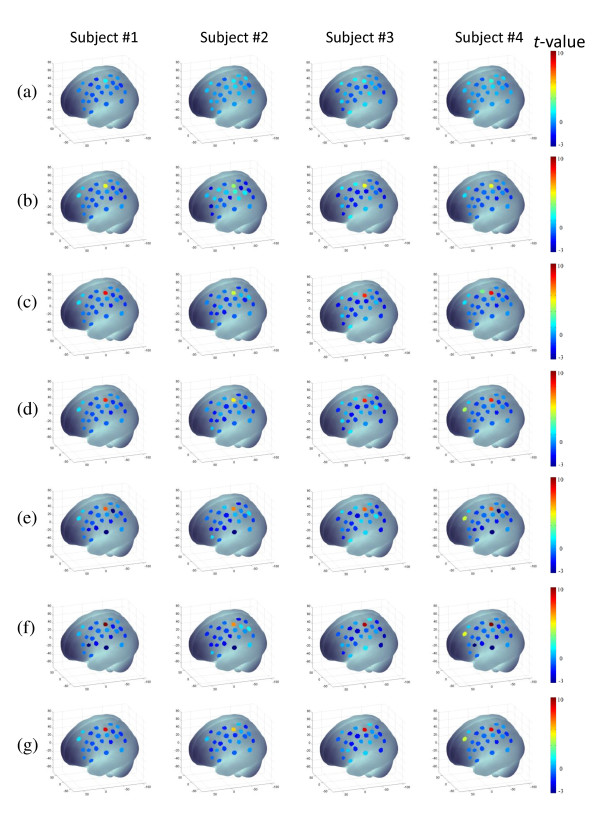
**Representative snapshots of the activation map at different times, for different subjects**. Rows (a) to (f) show *t*-images of the four subjects at different times (*T *= 120, 200, 300, 400, 500, and 552 sec). Row (f) shows *t*-images of four subjects estimated by the conventional GLM-based method. The results are presented by means of NFRI tools [47].

The motor cortex brain activity related to the finger-tapping task was found in 4 out of the 5 subjects. In the case of the fifth subject, there was no brain activation identified by the proposed framework. Figure 4 shows representative snapshots of the probability activation map (*t*-statistics map) at the different times *T *= 120, 200, 300, 400, 500, and 552 sec, from the top row (a) to the bottom row (f) of the detected area, for subjects 1 to 4. Row (g) shows the activation map estimated by the conventional method (the off-line GLM-based method, ordinary least squares followed by *t*-statistics). The *p*-value set for an individual test was *p*_*in *_< 0.05. At an early stage, around 120 sec, the proposed method could track the finger-tapping-related brain-active area, as indicated in Table 1 column 3. With Bonferroni correction, the time for the method to track the brain-active area was delayed by around 60 sec, as shown in Table 1 column 4. The estimated anatomical location of channel 6 was Broadmann area 4 (primary motor cortex), and of channel 4, Broadmann area 8. The result estimated by our framework was compared with the result estimated by the classical method. We found that at the final stage of the experiment, our results were almost consistent with those estimated by the conventional method. It is noteworthy that our method was able to locate and track the brain-active areas while the experiment was running and, thereby, to provide feedback on-line.

**Table 1:** Time and location of the brain activities tracked

Subject	Channel (location)	Time [sec] (*p_in _*< 0.05)	Time [sec] (*p_b_*, with Bonferroni correction)
1	6	118	177

2	6	115	170

3	6	129	186

4	4	335	402
	
	6	125	184

## Discussion

Changes in cerebral oxygenation reflect cerebral functional activity. In the present work, an updated version of the GLM was devised and used for on-line brain activation mapping. To demonstrate this framework, a finger-tapping task activation mapping study was carried out. This study allowed us to highlight several important features of our framework.

An obvious advantage of our method is its real-time applicability. The Kalman estimator has an acceptable computational overload, which allowed us to implement this method using Matlab in real-time (1.81 Hz in this study). The proposed method displays an updated brain activation map while the experiment is running. The finger-tapping-related brain-active area can be identified and tracked at the early stage of an experiment. The data analysis stage in the classical GLM framework can be conducted only after an experiment, and thus needs extra time. By contrast, with the proposed method, the model coefficients are recursively estimated, and the brain activation map can be updated at each time step. Accordingly, the method is able to track the brain-active area in an ongoing experiment, and provide an early warning to the experimenter when the subject is not responding appropriately or the system is not working properly or well. Thus, both the subject/patient and researcher can receive feedback in real-time.

**Figure 5:**
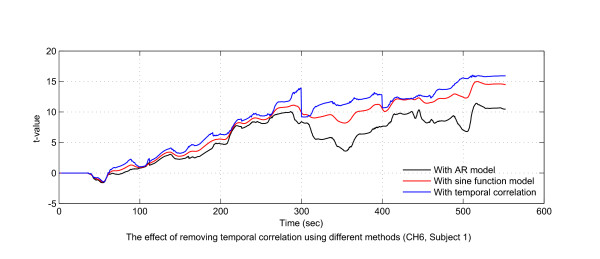
**Effect of temporal correlation reduction in NIRS time series**. The time course of inflated *t*-values caused by temporal correlation is shown in the figure. The effects of two different methods (the sine function model and the AR model) in reducing the temporal correlation are compared.

There are several physiological noises in NIRS data, including noises caused by cardiac and respiratory activity and blood pressure (Mayer wave) fluctuations, which might cause temporal correlation in the form of inflated *t*-values [27]. Ignoring these noises can lead to overestimation of brain activity. In [24,37], sine functions at different frequencies were used to model these noises; resultantly, some nonlinear terms were added to the classical GLM, and an extended Kalman filter was used to update the magnitude and phase shift of the noise terms. In the proposed framework, we used an AR(1) model to reduce the temporal correlation. We compared the effect of temporal correlation reduction using the two methods. Figure 5 indicates that both methods were able to reduce the temporal correlation in the NIRS time series and, moreover, that the AR(1) model performed better.

**Figure 6:**
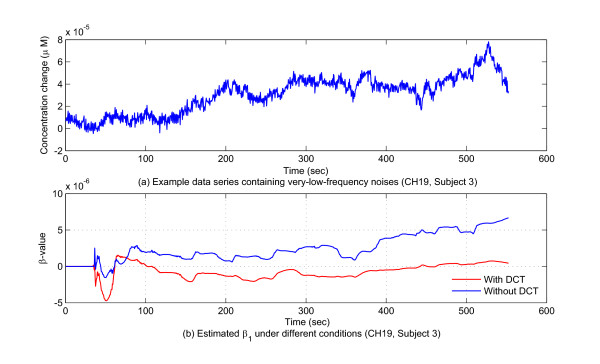
**Very-low-frequency noises and the DCT filter effect**. Panel (a) gives an example of a piece of NIRS data containing very-low frequency noises. Panel (b) shows the DCT filter preventing very-low frequency noises from leading to biased results.

Finally, the GLM combined with the Kalman filtering allowed for avoidance of very- low-frequency noises in the process of real-time estimation. In NIRS time series, very-low-frequency noises caused by optodes shifts or possible slow cardiac/respiratory artifacts sometimes exist. The upper plot in Figure 6 provides a sample data series containing very-low-frequency noise. The data in this plot was recorded at channel 19, subject 3. This channel covered the area of Bordemann area 9, where no brain activity was expected to be found in the current experiment. A clear slow shift of the baseline level can be identified by the human eye. In this study, a set of high-pass filters (discrete cosine transform, DCT) was modelled as regressors in the design matrix to remove the very-low-frequency noises on-line. The lower plot in Figure 6 shows estimation results using the DCT filter in comparison with those without using the DCT filter. This makes clear that without using the DCT filter, the estimation results were severely distorted by the low-frequency noise, which therefore would lead to a biased statistical analysis result.

There are some limitations of our method. First, the design matrix in the GLM was designed before the experiment. Different subjects might have individual hemodynamic responses. However, we assumed a general hemodynamic response model for different subjects in our framework. Our method might not work properly if a notable difference exists between the hemodynamic response of a subject and our modelled one. In [38], a set of linear combined Gaussian-based temporal functions was used to model the hemodynamic response. This model can approximate complex hemodynamic responses among different subjects, by means of multiple overlapping Gaussian functions. However, a recent work [39] suggested that event-evoked hemodynamic responses were similar among different subjects. In the current study, we also found a similarity in the hemodynamic responses, among all of our subjects. Therefore, we consider that it was sufficient to use the general hemodynamic model for estimation in current study.

The second limitation lies in the fact that we made only a preliminary conclusion about the feasibility of the proposed on-line framework, based on the results from 5 subjects. In the current study, brain activity at the primary motor cortex was found in 4 out of the 5 subjects investigated using the proposed framework. There was no brain activation area identified for subject 5, because the measurement noises (electric voltage fluctuations) induced by the equipment were very large in the case of this subject. Thus, no brain-active area could be identified based on the measured data. In order to produce a complete picture of the feasibility of the proposed framework, data needs to be measured for a greater number of subjects.

The *t*-statistics from the current study are based on the estimation of mean square error (MSE) via Equation (14). Therefore, while the experiment is running, the cumulative type I error increases as the test number grows. Thus, a post-hoc analysis is also needed to guarantee the statistical significance. We used Bonferroni correction for post-hoc analysis at each step in the current study. The *p_in _*for an individual test was set to 0.05, and the *p_b _*after Bonferroni correction was calculated as *p_b _*= (1 - (1 - *p_in_*)*^j^*]/*j*, where *j *represents the number of tests. As indicated in Table 1 column 4, the post-hoc analysis reduced the performance of the proposed framework, though it still yields promising results. Nevertheless, it might not be suitable for long-duration experiments (e.g. 6 hours' measurement for sleep studies). When the number of tests increases to a large value, the *p_b _*might be difficult to satisfy, resulting in an underestimation of brain activation. This is one of the limitations of the proposed framework. The problem probably can be avoided, either by reducing the sampling frequency over a long-duration measurement, thereby reducing the number of tests, or by controlling the experimentation time.

The time needed for locating different brain-active areas differed in the current study. Because Kalman filtering is a recursive process, the new information will be added as it arrives at each sampling time. Therefore, the estimation results from early stages are less reliable than those from later ones. In another aspect, the brain is a heterogeneous and dynamic organ that can process parallel work (in different brain areas), and so the same estimation process might perform differently for different brain areas. In our study, channel 4 of subject 4 was identified as a brain-active area during the finger-tapping task. The area covered by channel 4 is Brodmann area 8, responsible for voluntary eye movement, which is not expected to be active during finger tapping. This particular result might be accounted for simply as an indication that subject 4 was moving his eyes frequently while tapping his finger.

The DYNOT equipment in the present study employs a brush-less DC servomotor to provide for light source beam positioning [40]. The motor is driven by a controller containing freely programmable microprocessor. In this experiment, only a simple open-loop control scheme was designed for controlling the motor moving. Some complex control algorithms can be applied to achieve a more precise motor controlling [41-46].

One further issue in NIRS topographical applications is the fact that only a small portion of a detected area is measurable with sparsely distributed channels. Either tomographical NIRS detection or application of interpolation techniques might help increase it [20]. Both solutions would require extra processing time. Therefore, the issue of the development of a proper means of achieving higher spatial resolution in real-time applications also will have to be resolved in future research.

## Conclusions

A new Kalman-estimator- and GLM-based NIRS data analysis framework was demonstrated for real-time imaging of brain activity. In an experiment, this framework allowed for updating of the topographic activation map of the detected brain area, and, additionally, it could locate the activated brain area at an early stage by analyzing the noise-containing raw data.

## Competing interests

The authors declare that they have no competing interests.

## Authors' contributions

XSH performed the experiment and carried out the data processing. KSH participated in the data processing and revised the manuscript. SSG participated in theoretical aspects of the study design and evaluated the experiment. MYZ evaluated the experiments and gave suggestions to the manuscript. All of the authors read and approved the final manuscript.
